# Annotations

**Published:** 1910-08-20

**Authors:** 


### Annotations.

### School Inspection in Australia.

The inspection of school children is a new thing in Victoria, but has already revealed much. Dr. Harvey Sutton, in a lecture to secondary teachers some time ago, remarked that a good proportion of those children found with defects had been placed under medical treatment at once by their parents. Of over four thousand cards of questions to the parents not more than one hundred were missing. The average chest measurement was below the English average, but Australia was evolving a physical type quite different from those of England, Scotland, Ireland, or America. Mr. L. A. Adamson, headmaster of one of the large public schools for boys, said that in his own experience he found often that State school scholars on joining the school were already exhausted mentally and physically after the hard work necessary to win their scholarships. In the circumstances it seems imperative that medical inspection of school children in Australia shall be vigorously supported.

### The Law and Occultism in France.

Paragraph 7 of Article 479 of the French penal code provides punishment, in the shape of a fine of 11 to 15 francs, for those persons who carry out the profession of diviners, fortune-tellers, or of explainers of dreams. Article 405 of the same code deals with the offence of swindling, and empowers the tribunals to sentence to imprisonment and to inflict a fine of 50 to 3,000 francs, without prejudice to other accessory penalties. It might be thought that these laws provide sufficient safeguards against the machinations of medical charlatans of all kinds, not to mention such minor practitioners as magnetisers, mediums, *et hoc genus omne*, and no doubt they would prove efficacious if interpreted in the sense in which probably they were framed. But a change seems to have come over the French judicial authorities in view of a number of decisions lately given in several French courts with reference to prosecutions of practitioners of these sciences. The first case concerned the will of an English lady who for several years had resided in Paris, and who left a legacy to a medium, a young woman with whom she had been on terms of the greatest intimacy. The daughter of the testatrix disputed the will on the plea of undue influence, but lost the case, the Court maintaining that no doubt could be entertained on the subject of the existence of spiritualistic phenomena even if incapable of explanation, and that no evidence was forthcoming to show that the testatrix was otherwise than in full possession of her faculties when she made the will. It would thus seem that spiritism is in future to be raised to the level of a religion—at any rate in the eyes of the French law. It is remarkable, however, that at about the same time a correctional tribunal acquitted a notorious quack known as "Le Zouave Jacob," who was prosecuted for the illegal practice of medicine. This again is new jurisprudence, for the rascal had been previously convicted. Again, it is but yesterday that a female magnetiser was acquitted of a similar charge by the tribunal of Versailles on the ground that judgment could only be pronounced on questions of fact, and that it was no part of the judicial authorities to enter the domain of hypothetical science lest a set-back be administered to the evolution of that science towards infinite progress! The result of these characteristic pronouncements can only be the virtual abrogation of the penal enactments we have quoted above. It will henceforth be impossible to define where charlatinism ends and science begins. Provided that the quack or soothsayer steers clear of actual murder, and only administers remedies or advice which are harmless, he can laugh at the law *re* illegal practice, for he has but to point out that the progress of "science" is being hampered to obtain an acquittal, and—who knows?—perhaps even damages for malicious prosecution. In fact, he will henceforth enjoy an immunity comparable to that of English and American members of the brotherhood.

### An Medico Barba?

The question which has been discussed for some three hundred years, as to whether the doctor, and more especially the surgeon, should sacrifice that playground of microbes—his beard—is once more to the fore, especially in France. It is more than probable that everyone interested will act according to his own particular fancy in this matter, and it will probably be a long time before our Gallic *confrères* will be forced to sacrifice the glories of their facial decorations on the altar of hygiene. At one time, however, there was no choice in the matter, for Alma Mater commanded and everyone had to obey. On March 25, 1599, at the instance of one Barthélemi Perdulcis, a member, the Faculty of Medicine of Paris ordained that its bachelors admitted on that day should shave their beards. The names of the victims of this order have been preserved to this day, and are given in *La Chronique Medicale*. A little later, before the same Faculty, a thesis was sustained entitled "An medico barba?" Unfortunately the conclusions arrived at have been lost in oblivion. The obstetricians of the *grand siècle*, and in particular Mauriceau, were of opinion that accoucheurs, at least, ought to wear full beards to counteract the dangerous effects of their seductive personalities upon their charming but too susceptible patients! Those who have embarked upon the present crusade attack the problem from a somewhat different standpoint, and although the bacteriology of the beard has not as yet been the object of very profound study, their ideas are not unreasonable. Still, without being as radical as they would like, is it not possible that the sanctity of the laws of hygiene can be respected by the use of ordinary measures of cleanliness, or, at most, an antiseptic beard-bath. For what is there to prevent hair and eyebrows following beards if these terrible shears are allowed unfettered play? Our contemporary shudders to think what the effect on the ladies might be!

